# Cortisol and adrenal androgens as independent predictors of mortality in septic patients

**DOI:** 10.1371/journal.pone.0214312

**Published:** 2019-04-04

**Authors:** Rocío De Castro, David Ruiz, Bernardo-Alio Lavín, Jose Ángel Lamsfus, Luis Vázquez, Coral Montalban, Gilberto Marcano, Raquel Sarabia, María Paz-Zulueta, Cristina Blanco, Miguel Santibáñez

**Affiliations:** 1 Anesthesiology Service, Sierrallana Hospital, Torrelavega, Spain; 2 Endocrinology Service, Marqués de Valdecilla University Hospital, Santander, Spain; 3 Clinical Analysis Service, Marqués de Valdecilla University Hospital, Santander, Spain; 4 Nursing Department, University of Cantabria-IDIVAL, Santander, Spain; 5 Global Health research group, University of Cantabria, Santander, Spain; Klinikum rechts der Isar der Technischen Universitat Munchen, GERMANY

## Abstract

**Objective:**

To determine the prognostic value of cortisol, Dehydroepiandrosterone (DHEA) and Dehydroepiandrosterone-sulfate (DHEAS), together with their ratios (cortisol/DHEA and cortisol/DHEAS), as independent predictors of mortality in septic patients.

**Methods:**

Prospective cohort study of 139 consecutive patients with a diagnosis of severe sepsis or septic shock. Adrenal hormones were determined within the first 24 hours of the septic process. To determine and compare the predictive ability of each marker for the risk of unfavorable evolution (in-hospital, 28-day and 90-day mortality), ROC (Receiver Operating Characteristic) curves were constructed and the area under the curve (AUC) was determined. As measures of association, adjusted odds ratios (OR) with their 95% confidence intervals (95%CI) were estimated by unconditional logistic regression. Cortisol, DHEA and DHEAS results were compared to lactate, CRP, SOFA and APACHE II Scores.

**Results:**

Cortisol showed the best predictive ability, with AUCs of 0.758, 0.759 and 0.705 for in-hospital mortality, and 28-day and 90-day mortality, respectively; whereas AUCs for 28 days mortality for SOFA and APACHE II scores, and other biomarkers studied, such as Lactate or CRP, were 0.644, 0.618, 0.643 and 0.647, respectively. Associations between high cortisol levels (>17.5 μg/dL) and mortality were strong and statistically significant for in-hospital and 28-day mortality: adjusted ORs 10.13 and 9.45 respectively, and lower for long term mortality (90 days): adjusted OR 4.26 (95% CI 1.34–13.56), p trend 0.014. Regarding adrenal androgens, only positive associations were obtained for DHEAS and most of these positive associations did not yield statistical significance. Regarding Cortisol/DHEA and cortisol/DHEAS ratios, they did not improve the predictive ability of cortisol. The only exception was the cortisol/DHEAS ratio, which was the best predictor of mortality at 90 days (AUC 0.737), adjusted OR for highest cortisol/DHEAS ratio values 6.33 (95%CI 1.77–22.60), p trend 0.002.

**Conclusion:**

Basal cortisol measured within the first 24 hours of the septic process was the best prognostic factor for in-hospital and 28-day mortality, even superior to the Sequential Organ Failure Assessment (SOFA) or Acute Physiology and Chronic Health Evaluation II (APACHE II) scores. The cortisol/DHEAS ratio was an independent predictor of long-term mortality.

## Introduction

Sepsis is an organic dysfunction caused by an unregulated host response to infection [1]. Currently, it is one of the main causes of admission to an intensive care unit, with a mortality rate of between 25–30%, rising to 40–50% in the case of septic shock [2]. Early identification of patients at a higher risk of death is a major challenge. Despite the existence of multiple risk scales, none of these are sufficiently accurate to predict mortality with adequate sensitivity and specificity [3]. The incorporation of new biomarkers is a fundamental task that can improve the predictive ability and the therapeutic intervention in septic patients.

The activation of the hypothalamic-pituitary-adrenal (HPA) axis is an essential component of the general adaptation to illness [4]. During sepsis, an up-regulation of cortisol and adrenal androgens, such as DHEA, has been described, but not of DHEAS [5]. The role of cortisol and adrenal androgens as prognostic factors in the septic patient has shown conflicting results [6–11]. On the other hand, given their immunomodulatory counteracting actions, it has been proposed that the increase in the cortisol/DHEA and cortisol/DHEAS ratios may represent novel prognostic markers in septic patients [5,7,10].

The aims of this study were to assess the prognostic value of a single determination of cortisol, DHEA, DHEAS and their ratios on Intensive Care Unit (ICU) admission in severe sepsis and septic shock patients, as well as to compare this with the prognostic value of a single determination of classical biomarkers, such as arterial lactate or C-reactive protein (CRP) on ICU admission, and to evaluate whether the addition of the same to SOFA could improve the prognostic accuracy of this severity score.

## Material and methods

### Study population

In a prospective cohort study conducted in the ICU of the Sierrallana Hospital in Torrelavega (Spain), we analyzed serum samples from 139 consecutive patients included in the first 24 hours of severe sepsis or septic shock diagnosis, between November 2011 and December 2017.

The inclusion criteria consisted of patients aged 18 years or older and admitted to the ICU within 24 hours after diagnosis of severe sepsis or septic shock, according to the 2001 International Sepsis Definitions Conference [12].

The exclusion Criteria were: patients who received corticosteroid treatment or who were treated with drugs that affect adrenal function within a six month period prior to admission; patients with known HPA axis disease or adrenal insufficiency (AI); patients with acute liver failure or chronic stage Child-Pugh B or C liver disease.

All patients included in the study were treated according to the recommendations of the Surviving Sepsis Campaign 2012 guidelines [13].

### Serum hormone measurements

Basal cortisol, DHEA and DHEAS levels were determined between 8:00 and 09:00 a.m. All samples were processed within one hour of extraction.

For the determination of total cortisol, the chemiluminescent immunoassay of microparticles ARCHITECT (Abbot, Wiesbaden, Germany), was used, which has a sensitivity ≤ 1 (μg/dL) and a specificity of 0–0.9%, except for fludrocortisone (36.6%) and prednisolone (12.3%).

The levels of DHEA were quantified in serum using DRG-specific Radioimmunoassay (DRG Instruments, Marburg, Germany). Sensitivity: 0.06 ng/ml. Specificity: Very low cross reactivity <0.001% with DHEAS, isoandrosterone, androstenedione and other related steroids. Intra-assay reproducibility of the method is <3.8% and interassay reproducibility is <8.6%.

DHEAS levels were quantified in serum by competitive solid phase chemiluminescent enzyme immunoassay in a Siemens IMMULITE 2000 (Siemens Health Care Diagnostics, Gwynedd, UK). Sensitivity: 10 g/dl. Specificity: Cross reactivity <0.1% with related steroids. Intra-assay reproducibility of the method is 7.1% and interassay reproducibility is 9.8%.

### Additional measures

The APACHE II and SOFA scores were determined and samples were extracted for the determination of CRP, arterial lactate and other analyses, according to the routine in place at the unit.

More information at http://dx.doi.org/10.17504/protocols.io.ymxfu7n.

### Ethical approval and consent to participate

The study was conducted according to the guidelines of the Declaration of Helsinki and was approved by the Clinical Research Ethics Committee of Cantabria (CEIC: 2015–001), requiring signed informed consent from each patient or the responsible family member. This study was observational, and data for each patient were anonymous.

### Statistical analysis

For categorical variables, proportions were estimated using the Pearson chi-square test for comparisons or, alternatively, the exact Fisher test. For continuous variables, mean values with their standard deviation (SD) were estimated. The Student’s t-test or Mann-Whitney tests were used to analyze the relationship between quantitative variables and dichotomous categorical variables. The condition of normality was previously checked using the Shapiro-Wilk test.

To determine and compare the predictive capabilities of each biomarker and severity scores on the risk of unfavorable evolution (death), ROC (Receiver Operating Characteristic) curves were constructed and the area under the curve (AUC) was determined. Data were analyzed globally and restricted to patients with low albumin levels (<2.5 g/dl).

To estimate the strength of associations, the biomarkers and severity scores were divided into dichotomous variables (low versus high values) according to the median, and adjusted odds ratios (OR) with their 95% confidence intervals (95%CI) for mortality were calculated using unconditional logistic regression. The following potential confounders were pre-established to be included in the models: age (as continuous variable), sex, SOFA score (as a continuous variable) and diagnosis of severe sepsis or septic shock. In addition, exposure-response trends (biological gradient) were estimated using a logistic regression model with all potential confounders, categorizing the prognostic factors according to tertiles. The category below was the reference category except for DHEA and DHEAS.

The statistical analysis was performed using SPSS statistical software package version 22.0 and Stata 13.0 for Windows. The level of statistical significance was set at 0.05 and all tests were two-tailed.

## Results

Baseline characteristics are shown in Table 1. A total of 139 patients were included (61.9%, N = 86 men; 38.1%, N = 53 women). The mean age was 67.15±14.27 years and 33.1% had septic shock at admission. Twenty-five (18%) patients died in hospital, twenty-three (16.5%) at 28 days and thirty-one (21.6%) at 90 days. Deaths among female and older patients were more frequent and women were on average 4.89 years older than men, 95%CI (0.01 to 9.77), p = 0.049 (data not shown in tables).

**Table 1:** Baseline characteristics, in relation to in-hospital mortality.

*Variables *	*Total*	*Survivors*	*Non survivors*	*p*
	*(n = 139)*	*(n = 114)*	*(n = 25)*	
***Age*, *mean (SD)***	67.15 (14.27)	65.96 (14.54)	72.60 (11.79)	*0*.*035*
***Sex male (M)*, *female (F)*, *n(%)***	M: 86 (61.9)	M:75 (65,8)	M:11 (44)	*0*.*042*
	F: 53 (38.1)	F:39 (34,2)	F:14 (56)	
***Charlson comorbidity index*, *median (IQR)***	1.00 (0.00–3.00)	1.00 (1.00–3.00)	2.00 (0.50–2.50)	*0*.*272*
***Severe sepsis*, *n (%)***	93 (66.9)	79 (69.3)	14 (56)	*0*.*201*
***Septic shock*, *n (%)***	46 (33.1)	35 (30.7)	11 (44)	
***Source of infection***				*0*.*148*
Abdominal, n (%)	51 (36.7)	40 (35.1)	11 (44)	
Lung, n (%)	35 (25.2)	27 (23.7)	8 (32)	
Urogenital, n (%)	32 (23)	31 (27.2)	1 (4)	
Primary septicemia, n (%)	11 (7.9)	9 (7.9)	2 (8)	
Others, n (%)	10 (7.2)	7 (6.1)	3 (12)	
***APACHE II score*, *median (IQR)***	18.00 (12.00–22.00)	17.50 (11.00–21.00)	18.00 (14.50–23.00)	*0*.*342*
***SOFA*, *median (IQR)***	6.00 (4.00–7.00)	6.00 (3.00–7.00)	7.00 (4.00–9.00)	*0*.*032*
***Unit of hospitalization***				*0*.*008*
Medical, n (%)	102 (73.4)	89 (78.1)	13 (52)	
Surgical, n (%)	37 (26.6)	25 (21.9)	12 (48)	
***CRP (mg/L)*, *median (IQR)***	206.50 (133.80–280.70)	185.15 (125.10–263.87)	304.40 (156.50–369.90)	*0*.*005*
***Arterial lactate (mmol/L)*,** ***median (IQR)***	1.60 (1.20–2.40)	1.50 (1.17–2.25)	1.80 (1.35–5.10)	*0*.*029*
***ACTH (pgr/mL)*, *median (IQR)***	19.00 (13.00–40.00)	18.00 (11.50–34.50)	21.00 (14.00–53.00)	*0*.*248*
***Cortisol (μg/dL)*, *median (IQR)***	17.50 (13.10–26.00)	16.25 (12.72–22.85)	27.50 (17.45–41.45)	*<0*.*001*
***DHEA (ngr/mL)*, *median(IQR)***	2.70 (1.80–4.40)	2.70 (1.80–4.15)	2.80 (1.90–4.90)	*0*.*530*
***DHEAS (μg/dL)*, *median (IQR)***	54.30 (22.70–112.00)	56.00 (22.15–118.25)	38.80 (26.75–67.10)	*0*.*384*
***Cortisol/DHEA, (μg·dl⁻^1^/ng·ml⁻^1^) median (IQR)***	6.29 (4.28–9.61)	5.82 (4.07–8.96)	8.44 (5.31–14.09)	*0*.*014*
***Cortisol/DHEAS*,** (***ng/ng)*,** ***median (IQR)***	0.33 (0.17–0.79)	0.27 (0.16–0.68)	0.69 (0.34–1.09)	*0*.*003*
***Mechanical ventilation (No)*, *n (%)***	114 (82)	99 (86.8)	15 (60)	*0*.*002*
***Mechanical ventilation (Yes)*, *n (%)***	25 (18)	15 (86.8)	10 (40)	
Mechanical ventilation time days, mean (SD)	8.68 (7.59)	5.85 (4.96)	11.75 (8.88)	*0*.*050*
***Vasopressors (No)*, *n (%)***	81 (58,3)	68 (59.6)	13 (52)	*0*.*482*
***Vasopressors (Yes)*, *n (%)***	58 (41.7)	46 (40.4)	12 (48)	
Vasopressors time days, mean (SD)	3.33 (4.46)	2.61 (2.14)	5.06 (7.41)	*0*.*198*
***ICU length of stay*, *days mean (SD)***	8.57 (20.05)	7.75 (21.56)	11.42 (13.38)	*0*.*371*
***Hospital length of stay*,** ***days mean (SD)***	22.76 (24.40)	23.00 (26.36)	21.90 (16.14)	*0*.*826*

Among the prognostic factors studied, the best rate of prediction of in-hospital mortality was cortisol in both the total sample and in patients with albumin less than 2.5 g/dL. (AUCs 0.758 and 0.772) (Table 2). On the other hand, the predictive ability of adrenal androgens (DHEA and DHEAS) was lower regarding cortisol, AUCs 0.460 and 0.556 (Table 2). The ROC curve for SOFA, APACHE II, lactate and CRP yielded AUCs of 0.636, 0.561, 0.639 and 0.682, respectively (S1 Table). Adding cortisol to the logistic model increased the predictive capacity in relation to SOFA for in-hospital mortality: SOFA alone (AUC 0.636), SOFA + Cortisol (AUC 0.759) (S1 Table).

**Table 2:** Area under curve (AUC) for the adrenal function biomarkers in relation to in-hospital mortality, in the overall population and restricted to patients with low albumin levels (<2.5 g/dl).

Adrenal Biomarkers	Total			Albumin <2.5 g/dl		
	AUC	(95%	CI)	AUC	(95%	CI)
***Cortisol (μg/dL)***	0.758	0.658	0.857	0.772	0.67	0.875
***DHEA (ng/ml)***	0.460	0.335	0.585	0.461	0.324	0.598
***DHEAS (μg/dL)***	0.556	0.441	0.671	0.539	0.411	0.668
***Cortisol/DHEA (μg·dl⁻^1^/ng·ml⁻^1^)***	0.658	0.539	0.777	0.658	0.528	0.788
***Cortisol/DHEAS*** (***ng/ng)***	0.689	0.583	0.796	0.672	0.552	0.792

Regarding 28-day mortality, the AUC value for Cortisol was 0.759, higher than DHEA and DHEAS (AUCs 0.459 and 0.565) (S2 Table). SOFA and APACHE II AUCs were 0.644 and 0.618; and Lactate and CPR AUCs were 0.643 and 0.647, respectively (S3 Table).

Regarding 90-day mortality, similar AUC values were obtained: 0.705 for Cortisol (S4 Table), and 0.614, 0.612, 0.611 and 0.677, SOFA, APACHE II, Lactate and CPR respectively (S5 Table). The predictive ability of adrenal androgens for both DHEA and DHEAS was again lower when compared to cortisol during this period (S4 Table)

Tables 3, 4 and 5 show the strength of associations (OR) and exposure-response trends (Biological gradient) between adrenal function biomarkers in relation to in-hospital mortality and mortality at 28 and 90 days, respectively. In the regression analysis, the basal cortisol also revealed the strongest association when values were dichotomized according to the median, in relation to in-hospital, 28-day and 90-day mortality, with adjusted ORs of 3.80, 3.24 and 2.37, respectively (Tables 3, 4 and 5). For in-hospital mortality, a significant exposure-response trend was obtained: adjusted OR at the highest tertile (ORa T3) 10.13, 95%CI 2.05–50.04, p trend 0.003 (Table 3). A similar association was found for 28-day mortality: ORa T3 9.45, 95%CI 1.87–47.72, p trend 0.005 (Table 4). In relation to 90-day mortality, the association was lower, although preserving the exposure-response pattern: ORa T3 4.26, 95%CI 1.34–13.56, p trend 0.014 (Table 5).

**Table 3:** Crude and adjusted odds ratios for adrenal function biomarkers on the risk of in-hospital mortality (all-causes).

		Survivors	Non survivors						
Adrenal biomarkers	*Cut-of points*	*N = 114*	*N = 25*	*OR*	*(95%*	*CI)*	*ORa*	*(95%*	*CI)*
***Cortisol (μg/dL) (Median)***									
Low (reference)	< = 17.5	65	6	1.00	—		1.00	—	
High	17.6+	49	19	4.20	1.56	11.30	3.80	1.34	10.79
***Cortisol (μg/dL) (Tertiles)***									
Low (reference)	< = 14.4	46	2	1.00	—		1.00	—	
Medium	14.5–22.2	37	8	4.97	0.99	24.85	6.86	1.25	37.52
High	22.3+	31	15	11.13	2.38	52.13	10.13	2.05	50.04
*p linear trend*				*p = 0*.*001*			*p = 0*.*003*		
***DHEA (ng/ml)(Median)***									
High (reference)	2.8+	53	13	1	—		1	—	
Low	< = 2.7	61	12	0.80	0.22	1.88	0.88	0.34	2.25
***DHEA (ng/ml)(Tertiles)***									
High (reference)	3.5+	34	10	1	—		1	—	
Medium	2.3–3.4	37	7	0.64	0.22	1.88	0.57	0.18	1.83
Low	< = 2.2	43	8	0.63	0.23	1.78	0.72	0.23	2.23
*p linear trend*				*p = 0*.*385*			*p = 0*.*569*		
***DHEAS (μg/dl)(Median)***									
High (reference)	54.4+	60	9	1	—		1	—	
Low	< = 54.3	54	16	1.98	0.81	4.84	1.65	0.63	4.31
***DHEAS (μg/dl)(Tertiles)***									
High (reference)	79.1+	41	5	1	—		1	—	
Medium	33.7–79.0	35	11	2.58	0.82	8.13	1.81	0.52	6.33
Low	< = 33.6	38	9	1.94	0.60	6.31	1.63	0.46	5.76
*p linear trend*				*p = 0*.*315*			*p = 0*.*499*		
***Cortisol/DHEA, (μg·dl⁻^1^/ng·ml⁻^1^) (Median)***									
Low (reference)	< = 6.296	62	8	1.00	—		1.00	—	
High	6.297+	52	17	2.53	1.01	6.34	2.72	1.02	7.29
***Cortisol/DHEA (μg·dl⁻^1^/ng·ml⁻^1^) (Tertiles)***									
Low (reference)	< = 5.093	42	4	1.00	—		1.00	—	
Medium	5.094–8.292	39	8	2.15	0.60	7.72	2.31	0.59	9.12
High	8.293+	33	13	4.14	1.23	13.87	4.47	1.18	16.86
*p linear trend*				*p = 0*.*017*			*p = 0*.*023*		
***Cortisol/DHEAS (ng/ng) (Median)***									
Low (reference)	< = 0.326	64	6	1.00	—		1.00	—	
High	0.327+	50	19	4.05	1.51	10.90	3.59	1.26	10.28
***Cortisol /DHEAS (ng/ng) (Tertiles)***									
Low (reference)	< = 0.202	43	4	1.00	—		1.00	—	
Medium	0.203–0.608	38	8	2.26	0.63	8.12	1.96	0.51	7.54
High	0.609+	33	13	4.23	1.26	14.19	3.21	0.88	11.73
*p linear trend*	* *			* p = 0*.*016*			* p = 0*.*072*		

ORa: Odds ratio adjusted by age, sex, SOFA score and diagnosis of severe sepsis or septic shock.

**Table 4:** Crude and adjusted odds ratios for adrenal function biomarkers on the risk of 28-day mortality (all-causes).

* *	* *	*Survivors*	*Non survivors*		* *	* *	* *	* *	* *
* Adrenal biomarkers*	*Cut-off points*	*N = 116*	*N = 23*	*OR*	*(95%*	*CI)*	*ORa*	*(95%*	*CI)*
***Cortisol (μg/dL) (Median)***									
*Low (reference)*	< = 17.5	65	6	1.00	—	—	1.00	—	—
*High*	17.6+	51	17	3.61	1.33	9.82	3.24	1.12	9.40
***Cortisol (μg/dL) (Tertiles)***									
*Low (reference)*	< = 14.4	46	2	1.00	—	—	1.00	—	—
*Medium*	14.5–22.2	38	7	4.24	0.83	21.61	6.48	1.13	37.05
*High*	22.3+	32	14	10.06	2.14	47.35	9.45	1.87	47.72
*p linear trend*				*p = 0*.*001*			*p = 0*.*005*		
***DHEA (ng/ml)(Median)***									
*High (reference)*	2.8+	54	12	1.00	—	—	1.00	—	—
*Low*	< = 2.7	62	11	0.80	0.33	1.95	0.89	0.33	2.38
***DHEA (ng/ml)(Tertiles)***									
*High (reference)*	3.5+	34	10	1.00	—	—	1,00	—	—
*Medium*	2.3–3.4	39	5	0.44	0.14	1.40	0.35	0.10	1.27
*Low*	< = 2.2	43	8	0.63	0.22	1.78	0.72	0.23	2.30
*p linear trend*				*p = 0*.*384*			*p = 0*.*575*		
***DHEAS (μg/dl)(Median)***									
*High (reference)*	54.4+	61	8	1.00	—	—	1.00	—	—
*Low*	< = 54.3	55	15	2.08	0.82	5.28	1.70	0.62	4.64
***DHEAS (μg/dl)(Tertiles)***									
*High (reference)*	79.1+	42	4	1.00	—	—	1.00	—	—
*Medium*	33.7–79.0	35	11	3.30	0.96	11.28	2.16	0.56	8.25
*Low*	< = 33.6	39	8	2.15	0.60	7.72	1.73	0.44	6.80
*p linear trend*				*p = 0*.*287*				*p = 0*.*513*	
***Cortisol/DHEA, (μg·dl⁻^1^/ng·ml⁻^1^) (Median)***									
*Low (reference)*	< = 6.296	62	8	1.00	—	—	1.00	—	—
*High*	6.297+	54	15	2.15	0.85	5.47	2.40	0.87	6.63
***Cortisol/DHEA (μg·dl⁻^1^/ng·ml⁻^1^) (Tertiles)***									
*Low (reference)*	< = 5.093	43	3	1,00	—	—	1.00	—	—
*Medium*	5.094–8.292	39	8	2.94	0.73	11.87	3.74	0.81	17.3
*High*	8.293+	34	12	5.06	1.32	19.37	6.40	1.41	29.01
*p linear trend*				*p = 0*.*015*			*p = 0*.*015*		
***Cortisol/DHEAS (ng/ng) (Median)***									
*Low (reference)*	< = 0.326	65	5	1.00	—	—	1.00	—	—
*High*	0.327+	51	18	4.59	1.60	13.20	4.21	1.36	13.05
***Cortisol /DHEAS (ng/ng) (Tertiles)***									
*Low (reference)*	< = 0.202	44	3	1.00	—	—	1.00	—	—
*Medium*	0.203–0.608	38	8	3.09	0.76	12.47	2.81	0.64	12.38
*High*	0.609+	34	12	5.18	1.35	19.81	3.98	0.95	16.72
*p linear trend*			* *	* p = 0*.*013*	* *	* *	* p = 0*.*060*	* *	

ORa: Odds ratio adjusted by age, sex, SOFA score and diagnosis of severe sepsis or septic shock.

**Table 5:** Crude and adjusted odds ratios for adrenal function biomarkers on the risk of 90-day mortality (all-causes).

		Survivors	Non survivors						
Adrenal biomarkers	*Cut-off points*	*108*	*N = 31*	*OR*	*(95%*	*CI)*	*ORa*	*(95%*	*CI)*
***Cortisol (μg/dL) (Median)***									
Low (reference)	< = 17.5	61	10	1.00	—		1.00	—	
High	17.6+	47	21	2.73	1.17	6.34	2.37	0.97	5.82
***Cortisol (μg/dL) (Tertiles)***									
Low (reference)	< = 14.4	43	5	1.00	—		1.00	—	
Medium	14.5–22.2	36	9	2.15	0.66	6.99	3.05	0.85	10.92
High	22.3+	29	17	5.04	1.67	15.19	4.26	1.34	13.56
*p linear trend*				*p = 0*.*003*			*p = 0*.*014*		
***DHEA (ng/ml)(Median)***									
High (reference)	2.8+	51	15	1.00	—		1.00	—	
Low	< = 2.7	57	16	0.95	0.43	2.12	1.13	0.47	2.71
***DHEA (ng/ml)(Tertiles)***									
High (reference)	3.5+	33	11	1.00	—		1.00	—	
Medium	2.3–3.4	36	8	0.67	0.24	1.86	0.63	0.21	1.92
Low	< = 2.2	39	12	0.92	0.36	2.36	1.13	0.40	3.23
*p linear trend*				*p = 0*.*890*			*p = 0*.*786*		
***DHEAS (μg/dl)(Median)***									
High (reference)	54.4+	60	9	1.00	—		1.00	—	
Low	< = 54.3	48	22	3.06	1.29	7.25	2.71	1.07	6.85
***DHEAS (μg/dl)(Tertiles)***									
High (reference)	79.1+	41	5	1.00	—		1.00	—	
Medium	33.7–79.0	34	12	2.89	0.93	9.03	2.05	0.60	7.04
Low	< = 33.6	33	14	3.48	1.14	10.65	3.01	0.91	10.00
*p linear trend*				*p = 0*.*032*			*p = 0*.*072*		
***Cortisol/DHEA, (μg·dl⁻^1^/ng·ml⁻^1^) (Median)***									
Low (reference)	< = 6.296	60	10	1.00	—		1.00	—	
High	6.297+	48	21	2.63	1.13	6.10	2.84	1.14	7.09
***Cortisol/DHEA (μg·dl⁻^1^/ng·ml⁻^1^) (Tertiles)***									
Low (reference)	< = 5.093	41	5	1.00	—		1.00	—	
Medium	5.094–8.292	38	9	1.94	0.60	6.31	2.13	0.59	7.72
High	8.293+	29	17	4.81	1.59	14.51	5.71	1.63	19.99
*p linear trend*				*p = 0*.*004*			*p = 0*.*004*		
***Cortisol/DHEAS (ng/ng) (Median)***									
Low (reference)	< = 0.326	64	6	1.00	—		1.00	—	
High	0.327+	44	25	6.06	2.30	15.99	5.93	2.08	16.88
***Cortisol /DHEAS (ng/ng) (Tertiles)***									
Low (reference)	< = 0.202	43	4	1.00	—		1.00	—	
Medium	0.203–0.608	38	8	2.26	0.63	8.12	2.10	0.55	8.07
High	0.609+	27	19	7.57	2.32	24.64	6.33	1.77	22.60
*p linear trend*	* *			* p = 0*.*000*	* *	* *	* p = 0*.*002*		

ORa: Odds ratio adjusted by age, sex, SOFA score and diagnosis of severe sepsis or septic shock.

Among the severity scores SOFA and APACHE II, crude positive non-significant associations (the higher the score, the higher risk of mortality) were obtained: for in-hospital mortality, crude OR at the highest tertile (OR T3) of APACHE II 1.79, 95%CI 0.58–5.51, p trend 0.319; OR T3 of SOFA 2.10, 95%CI 0.68–6.48, p trend 0.203 (S6 Table). However, after adjusting for age, gender, and severe sepsis or septic shock, these positive associations disappeared for APACHE II and diminished for SOFA (S6 Table). The same patterns were observed for mortality at 28 and 90 days (S7 and S8 Tables).

Regarding Lactate and CRP, the ORa T3 of lactate was 1.26, 95%CI 0.37–4.24, p trend 0.712; and the ORa T3 of CRP was 2.70, 95%CI 0.85–8.56, p trend was 0.074 in relation to in-hospital mortality (S6 Table). Similar associations were obtained for mortality at 28 and 90 days (S7 and S8 Tables).

Regarding adrenal androgens, DHEA and DHEAS, only positive associations were obtained for DHEAS in any of the studied mortality periods. Most of these positive associations did not yield statistical significance (Tables 3–5).

Regarding Cortisol/DHEA and cortisol/DHEAS ratios, these ratios were also associated with statistically significant increases in mortality risk and exposure response patterns. However, these associations were lower than associations for cortisol alone in relation to “in-hospital and at 28-day mortality” (Tables 3 and 4). On the contrary, for 90-day mortality, the associations for both ratios were greater than those for cortisol alone: ORa T3 of cortisol/DHEA ratio 5.71, 95%CI 1.63–19.99, p trend 0.004; ORa T3 of cortisol/DHEAS ratio 6.33, 95%CI 1.77–22.60, p trend 0.002 (Table 5).

## Discussion

Adrenal hormones play a key role in adaptation to stress. Our results support that basal cortisol, measured in the first 24 hours after diagnosis of sepsis, is an important independent prognostic factor for both short (in-hospital) and medium term mortality (28 days). However, the cortisol/DHEAS ratio would have a higher predictive capacity to cortisol alone for long-term mortality (90 days).

The associations between cortisol levels above the median (>17.6 μg/dl) and the increase of in-hospital and 28-day mortality remained after adjusting for the main confounding variables, such as age, sex, SOFA scale value and severe sepsis or septic shock status, supporting the independence of this association. In addition, an exposure-response pattern was obtained, where higher levels revealed a greater risk of mortality. Patients with high levels (third tertile) had about a ten-fold risk of in-hospital 28-day mortality compared with patients with lower levels of cortisol. This suggests causality, as both the independence of the association, and the strength and exposure-response pattern, are Bradford Hill's criteria for causality [14]. Likewise, the predictive capacity of a single cortisol determination, in relation to the AUC determined by the ROC curves, was superior to the more complex severity scores (SOFA and APACHE II), and to the classically used biomarkers (Lactate and CRP), to predict the risk of mortality in the septic patient. The predictive capacity in relation to SOFA, increased by adding cortisol to the logistic models. Thus, cortisol alone could be a useful biomarker to predict in-hospital and 28-day mortality with a greater predictive capacity than the SOFA and APACHE II scores and other biomarkers studied (lactate and CRP).

Our results are consistent with those described by other authors who have identified an association between elevated cortisol levels and an increased risk of mortality in septic patients [5–10,15]. The septic process has a dynamic course in which a balance between the hyperimmune response and immunosuppression is essential [16]. Cortisol has known anti-inflammatory and immunosuppressive actions, mostly triggered by its binding to a glucocorticoid receptor (GR) [17]. Although the complications of sepsis have been associated with an uncontrolled inflammatory response, this hypothesis is largely derived from work on animal models. In fact, the use of anti-inflammatory agents for the treatment of sepsis has not been shown to be beneficial in humans and may even cause harmful effects if used during the hypoimmune phase [18–20]. Therefore, an excessive increase in cortisol levels in the early stages of the septic process can destabilize the balance towards the hypoimmune state at an inappropriate time and have a negative effect on prognosis [21]. Moreover, it has recently been described how the use of glucocorticoids can decrease the ability of cortisol to bind to GR, which can lead to increased peripheral resistance to its actions [22].

In contrast to cortisol, adrenal androgens have immunostimulatory actions [23]. In our study, adrenal androgens had a lower prognostic capacity for in-hospital and 28-day mortality. Predictive capacity was superior for DHEAS in relation to DHEA. The predictive capacity of the cortisol/DHEA and cortisol/DHEAS ratios was also not greater than that of cortisol alone for short and medium-term mortality. However, concerning long-term mortality, the role for cortisol/adrenal androgens ratios could be greater, especially for the cortisol/DHEAS ratio, since the area under the curve for this index (AUC 0.74) was greater than that of cortisol itself (AUC 0.71).

Few studies have evaluated the role of adrenal androgens in sepsis. Mueller C et al., [7] determined that the values of cortisol/DHEA and cortisol/DHEAS ratios increased significantly in patients with more severe pneumonias. Marx C et al., [10] found lower levels of DHEAS at the beginning and end of sepsis among the non survivors. Arlt et al., [5] reported a dissociation between DHEA and DHEAS levels in septic patients and an increase in mortality associated with increased cortisol/DHEA ratio values.

During sepsis there is an increase in cortisol levels especially in relation to a decrease in its peripheral metabolism [24]. This prolonged sustained increase in cortisol can lead to inhibition of the HPA axis by negative feedback with the consequent risk of developing AI [25].

None of the tests used for the assessment of the integrity of the HPA axis is considered adequate in the early stages of the septic process, as these rely on the determination of serum cortisol and levels of the same are significantly influenced by changes in peripheral metabolism. DHEAS, the most abundant steroid hormone in the circulatory system, is almost entirely secreted by the reticular area of the adrenal cortex under the stimulus of ACTH. For these reasons, it has recently proven its usefulness as an indicator of the integrity of the HPA axis [26]. Our hypothesis is that DHEAS may be an appropriate tool for early diagnosis of septic patients at high risk of developing AI. Elevation of the cortisol/DHEAS ratio may identify patients with increased HPA axis suppression and prolonged loss of the trophic effect of ACTH on the adrenal glands, and thus increased long-term risk of AI. This may explain why the cortisol/DHEAS ratio is a good predictor of long-term mortality in our study. Further prospective studies specifically designed to test this hypothesis would be necessary.

Our study has some limitations. First, we used the determination of total cortisol, whose values are influenced by the levels of transport proteins. In our study, 77% of patients had an albumin level of less than 2.5 (g/dl). However, the predictive ability of cortisol remained constant in this group, supporting the validity of our results. Additionally, free cortisol analysis is a laborious technique performed in very few specialized centers and would therefore, be difficult to apply in routine clinical practice. Another limitation is that we only performed a single measurement. The changes in adrenal hormone values throughout the course of sepsis are well known and, therefore, our results can be reproducible if extraction is performed within the first 24 hours of the septic process. This study also presents important strengths such as its prospective nature, and the thoroughness of the inclusion criteria with a comprehensive exclusion of all patients who had received drugs that interfered with the HPA axis.

## Conclusions

In septic patients, serum total cortisol determined at 8–9 a.m. within the first 24 hours of the diagnosis, may be a useful biomarker for predicting in-hospital and 28-day mortality, with a greater predictive ability than the SOFA and APACHE II scores and other biomarkers studied, such as Lactate or CRP. The addition of adrenal androgens to perform the cortisol/androgens ratio would not increase predictive ability and, therefore, its determination for mortality restricted to in-hospital or 28-day periods does not seem clinically useful. Adrenal androgens, especially DHEAS, may have a greater role in long-term mortality. In this case, the predictive capacity of cortisol/DHEAS may be slightly higher than that of cortisol alone.

## Supporting information

S1 Table Area under the curve (AUC) of the rest of biomarkers and SOFA and APACHE II scores in relation to in-hospital mortality. (DOCX)

S2 Table Area under curve (AUC) for the adrenal biomarkers in relation to 28-day mortality, in the overall population and restricted to patients with low albumin levels (<2.5 g/dl). (DOC)

S3 Table Area under the curve (AUC) of the rest of biomarkers and SOFA and APACHE II scores in relation to 28-day mortality. (DOC)

S4 Table Area under curve (AUC) for the adrenal biomarkers in relation to 90-day mortality, in the overall population and restricted to patients with low albumin levels (<2.5 g/dl). (DOC)

S5 Table Area under the curve (AUC) of the rest of biomarkers and SOFA and APACHE II scores in relation to 90-day mortality. (DOC)

S6 Table Crude and adjusted odds ratios for the rest of biomarkers and severity scores on the risk of all-cause in-hospital mortality. (DOC)

S7 Table Crude and adjusted odds ratios for the rest of biomarkers and severity scores on the risk of all-cause 28-day mortality. (DOC)

S8 Table Crude and adjusted odds ratios for the rest of biomarkers and severity scores on the risk of all-cause 90-day mortality. (DOC)

## Abbreviations

ACTH: Adrenocorticotropic hormone
AI: Adrenal insufficiency
APACHE II: Acute Physiology And Chronic Health Evaluation II
AUC: Area under the curve
CEIC: Clinical research Ethics Committee of Cantabria
CI: Confidence intervals
CRP: C-reactive protein
DHEA: Dehydroepiandrosterone
DHEAS: Dehydroepiandrosterone-sulfate
GR: Glucocorticoid receptor
HPA: Hypothalamic-pituitary-adrenal
ICU: Intensive Care Unit
IQR: Interquartile range
OR: Odds ratios
ROC: Receiver Operating Characteristic
SD: Standard Deviation
SOFA: Sequential Organ Failure Assessment
