# Supplementary material for: Cortisol and adrenal androgens as independent predictors of mortality in septic patients
Source: PLoS One. 2019 Apr 4;14(4):e0214312. doi: 10.1371/journal.pone.0214312 (PMC6448869; doi:10.1371/journal.pone.0214312)
Supplement: S1 Table — (DOCX) [file pone.0214312.s001.docx]

**S1 Table. Area under the curve (AUC) of the rest of biomarkers and SOFA and APACHE II scores in relation to in-hospital mortality**.

| **Variables** | **AUC** | **(95%** | **CI)** |
| --- | --- | --- | --- |
| ***SOFA*** | **0.636** | 0.524 | 0.748 |
| ***APACHE*** | **0.561** | 0.441 | 0.680 |
| ***Lactate (nmol/L)*** | **0.639** | 0.513 | 0.765 |
| ***CRP (ng/ml)*** | **0.682** | 0.553 | 0.810 |
| ***SOFA + Cortisol (µg/dL)*** | **0.759** | 0.654 | 0.864 |

The values mentioned in the results section of the article are shown in bold type.
