# Supplementary material for: Cortisol and adrenal androgens as independent predictors of mortality in septic patients
Source: PLoS One. 2019 Apr 4;14(4):e0214312. doi: 10.1371/journal.pone.0214312 (PMC6448869; doi:10.1371/journal.pone.0214312)
Supplement: S2 Table — (DOC) [file pone.0214312.s002.doc]

S2 Table. Area under curve (AUC) for the adrenal biomarkers in relation to 28-day mortality, in the overall population and restricted to patients with low albumin levels (<2.5 g/dl).

| **Adrenal Biomarkers** | **Total** |  |  | **Albumin <2.5 g/dl** | |  |
| --- | --- | --- | --- | --- | --- | --- |
|  | **AUC** | **(95%** | **CI)** | **AUC** | **(95%** | **CI)** |
| ***Cortisol (µg/dL)*** | **0.759** | 0.652 | 0.866 | 0.775 | 0.665 | 0.886 |
| ***DHEA (ng/ml)*** | **0.459** | 0.327 | 0.591 | 0458 | 0.313 | 0.603 |
| ***DHEAS (µg/dL)*** | **0.565** | 0.452 | 0.677 | 0.546 | 0.42 | 0.673 |
| ***Cortisol/DHEA*** (***µg·dl⁻¹/ng·ml⁻¹)*** | 0.662 | 0.541 | 0.784 | 0.66 | 0.527 | 0.793 |
| ***Cortisol/DHEAS*** (***ng/ng)*** | 0.705 | 0.603 | 0.808 | 0.69 | 0.574 | 0.806 |

The values mentioned in the results section of the article are shown in bold type.
