# Supplementary material for: Cortisol and adrenal androgens as independent predictors of mortality in septic patients
Source: PLoS One. 2019 Apr 4;14(4):e0214312. doi: 10.1371/journal.pone.0214312 (PMC6448869; doi:10.1371/journal.pone.0214312)
Supplement: S3 Table — (DOC) [file pone.0214312.s003.doc]

S3 Table. Area under the curve (AUC) of the rest of biomarkers and SOFA and APACHE II scores in relation to 28-day mortality.

| **Variables** | **AUC** | **(95%** | **CI)** |
| --- | --- | --- | --- |
| ***SOFA*** | **0.644** | 0.533 | 0.755 |
| ***APACHE*** | **0.618** | 0.502 | 0.733 |
| ***Lactate (nmol/L)*** | **0.643** | 0.512 | 0.775 |
| ***CRP (ng/ml)*** | **0.647** | 0.513 | 0.78 |
| ***SOFA + Cortisol (µg/dL)*** | 0.770 | 0.661 | 0.880 |

The values mentioned in the results section of the article are shown in bold type.
