# Supplementary material for: Cortisol and adrenal androgens as independent predictors of mortality in septic patients
Source: PLoS One. 2019 Apr 4;14(4):e0214312. doi: 10.1371/journal.pone.0214312 (PMC6448869; doi:10.1371/journal.pone.0214312)
Supplement: S4 Table — (DOC) [file pone.0214312.s004.doc]

S4 Table. Area under curve (AUC) for the adrenal biomarkers in relation to 90-day mortality, in the overall population and restricted to patients with low albumin levels (<2.5 g/dl).

| **Adrenal Biomarkers** | **Total** |  |  | **Albumin <2.5 g/dl** | |  |
| --- | --- | --- | --- | --- | --- | --- |
|  | **AUC** | **(95%** | **CI)** | **AUC** | **(95%** | **CI)** |
| ***Cortisol (µg/dL)*** | **0.705** | 0.601 | 0.809 | 0.712 | 0.602 | 0.822 |
| ***DHEA (ng/ml)*** | 0.509 | 0.39 | 0.629 | 0.523 | 0.393 | 0.654 |
| ***DHEAS (µg/dL)*** | 0.633 | 0.526 | 0.741 | 0.631 | 0.512 | 0.749 |
| ***Cortisol/DHEA*** (***µg·dl⁻¹/ng·ml⁻¹)*** | 0.675 | 0.564 | 0.787 | 0.684 | 0.563 | 0.804 |
| ***Cortisol/DHEAS*** (***ng/ng)*** | **0.737** | 0.641 | 0.833 | 0.730 | 0.624 | 0.837 |

The values mentioned in the results section of the article are shown in bold type.
