# Supplementary material for: Cortisol and adrenal androgens as independent predictors of mortality in septic patients
Source: PLoS One. 2019 Apr 4;14(4):e0214312. doi: 10.1371/journal.pone.0214312 (PMC6448869; doi:10.1371/journal.pone.0214312)
Supplement: S5 Table — (DOC) [file pone.0214312.s005.doc]

**S5 Table. Area under the curve (AUC) of the rest of biomarkers and SOFA and APACHE II scores in relation to 90-day mortality.**

**The values mentioned in the results section of the article are shown in bold type.**

| **Variables** | **AUC** | **(95%** | **CI)** |
| --- | --- | --- | --- |
| ***SOFA*** | **0.614** | 0.507 | 0.722 |
| ***APACHE*** | **0.612** | 0.507 | 0.717 |
| ***Lactate (nmol/L)*** | **0.611** | 0.492 | 0.729 |
| ***CRP (ng/ml)*** | **0.677** | 0.568 | 0.787 |
| ***SOFA + Cortisol (µg/dL)*** | 0.717 | 0.607 | 0.827 |
