# Supplementary material for: Cortisol and adrenal androgens as independent predictors of mortality in septic patients
Source: PLoS One. 2019 Apr 4;14(4):e0214312. doi: 10.1371/journal.pone.0214312 (PMC6448869; doi:10.1371/journal.pone.0214312)
Supplement: S6 Table — (DOC) [file pone.0214312.s006.doc]

S6 Table. Crude and adjusted odds ratios for the rest of biomarkers and severity scores on the risk of all-cause in-hospital mortality.

|  |  | **Survivors** | **Non survivors** | |  |  |  |  |  |
| --- | --- | --- | --- | --- | --- | --- | --- | --- | --- |
| **Variables** | ***Cut-off points*** | ***N=114*** | ***N=25*** | ***OR*** | ***(95%*** | ***CI)*** | ***ORa*** | ***(95%*** | ***CI)*** |
| ***APACHEII (Median)*** |  |  |  |  |  |  |  |  |  |
| Low (reference) | <= 18 | 63 | 13 | 1.00 | -- |  | 1.00 | -- |  |
| High | 19+ | 51 | 12 | 1.14 | 0.48 | 2.71 | 0.57 | 0.21 | 1.51 |
| ***APACHEII (Tertiles)*** |  |  |  |  |  |  |  |  |  |
| Low (reference) | <= 14 | 43 | 6 | 1.00 | -- | -- | 1.00 | -- |  |
| Medium | 15 - 20 | 35 | 10 | 2.05 | 0.68 | 6.19 | 0.85 | 0.24 | 3.04 |
| High | 21+ | 36 | 9 | **1.79** | **0.58** | **5.51** | 0.52 | 0.13 | 2.04 |
| *p linear trend* |  |  |  | *p=0.319* |  |  | *p=0.311* |  |  |
| ***SOFA* (Median)*** |  |  |  |  |  |  |  |  |  |
| Low (reference) | <= 6 | 73 | 12 | 1.00 | -- | -- | 1.00 | -- | -- |
| High | 7+ | 41 | 13 | 1.93 | 0.81 | 4.62 | 1.45 | 0.56 | 3.73 |
| ***SOFA *(Tertiles)*** |  |  |  |  |  |  |  |  |  |
| Low (referene) | <= 4 | 44 | 7 | 1.00 | -- | -- | 1.00 | -- | -- |
| Medium | 5 – 7 | 46 | 10 | 1.37 | 0.48 | 3.91 | 1.19 | 0.39 | 3.58 |
| High | 8+ | 24 | 8 | **2.10** | **0.68** | **6.48** | 1.49 | 0.41 | 5.33 |
| *p linear trend* |  |  |  | *p=0.203* |  |  | *p=0.545* |  |  |
| ***Arterial lactate, mmol/L (Median)*** |  |  |  |  |  |  |  |  |  |
| Low (reference) | <= 1.6 | 63 | 10 | 1.00 | -- | -- | 1.00 | -- | -- |
| High | 1.7+ | 51 | 15 | 1.85 | 0.77 | 4.47 | 1.14 | 0.44 | 2.99 |
| ***Arterial lactate mmol/L (Tertiles)*** |  |  |  |  |  |  |  |  |  |
| Low (reference) | <= 1.3 | 42 | 6 | 1.00 | -- | -- | 1.00 | -- | -- |
| Medium | 1.4 - 2.0 | 39 | 8 | 1.44 | 0.46 | 4.51 | 1.14 | 0.34 | 3.87 |
| High | 2.1+ | 33 | 11 | 2.33 | 0.78 | 6.97 | **1.26** | **0.37** | **4.24** |
| *p linear trend* |  |  |  | *p=0.124* |  |  | *p=0.712* |  |  |
| ***CRP, mg/L (Median)*** |  |  |  |  |  |  |  |  |  |
| Low (reference) | <= 206.5 | 62 | 8 | 1.00 | -- | -- | 1.00 | -- | -- |
| High | 206.6+ | 52 | 17 | 2.53 | 1.01 | 6.34 | 2.10 | 0.79 | 5.57 |
| ***CRP, mg/L (Tertiles)*** |  |  |  |  |  |  |  |  |  |
| Low (reference) | <= 150.2 | 41 | 6 | 1.00 | -- | -- | 1.00 | -- | -- |
| Medium | 150.3 - 251.9 | 40 | 6 | 1.02 | 0.30 | 3.45 | 0.98 | 0.27 | 3.52 |
| High | 252.0+ | 33 | 13 | 2.69 | 0.92 | 7.85 | **2.70** | **0.85** | **8.56** |
| *p linear trend* |  |  |  | *p=0,057* |  |  | *p=0.074* |  |  |

ORa: Odds ratio adjusted by age, sex, SOFA score and diagnosis of severe sepsis or septic shock. The values mentioned in the results section of the article are shown in bold type.
