# Supplementary material for: Cortisol and adrenal androgens as independent predictors of mortality in septic patients
Source: PLoS One. 2019 Apr 4;14(4):e0214312. doi: 10.1371/journal.pone.0214312 (PMC6448869; doi:10.1371/journal.pone.0214312)
Supplement: S7 Table — (DOC) [file pone.0214312.s007.doc]

**S7 Table. Crude and adjusted o**dds ratios for the rest of biomarkers and severity scores on the risk of all-cause 28-day mortality.

|  |  | **Survivors** | **Non survivors** | |  |  |  |  |  |
| --- | --- | --- | --- | --- | --- | --- | --- | --- | --- |
| **Variables** | ***Cut-off points*** | ***N=116*** | ***N=23*** | ***OR*** | ***(95%*** | ***CI)*** | ***ORa*** | ***(95%*** | ***CI)*** |
| ***APACHEII (Median)*** |  |  |  |  |  |  |  |  |  |
| Low (reference) | <= 18 | 66 | 10 | 1.00 | -- |  | 1.00 | -- |  |
| High | 19+ | 50 | 13 | 1.72 | 0.70 | 4.23 | 0.90 | 0.33 | 2.48 |
| ***APACHEII (Tertiles)*** |  |  |  |  |  |  |  |  |  |
| Low (reference) | <= 14 | 45 | 4 | 1.00 | -- |  | 1.00 | -- |  |
| Medium | 15 – 20 | 36 | 9 | 2.81 | 0.80 | 9.88 | 1.15 | 0.28 | 4.72 |
| High | 21+ | 35 | 10 | 3.21 | 093 | 11.12 | 0.98 | 0.23 | 4.19 |
| *p linear trend* |  |  |  | *p=0.069* |  |  | *p=0.912* |  |  |
| ***SOFA* (Median)*** |  |  |  |  |  |  |  |  |  |
| Low (reference) | <= 6 | 75 | 10 | 1.00 | -- | -- | 1.00 | -- | -- |
| High | 7+ | 41 | 13 | 2.38 | 0.96 | 5.90 | 1.79 | 0.66 | 4.82 |
| ***SOFA *(Tertiles)*** |  |  |  |  |  |  |  |  |  |
| Low (referene) | <= 4 | 45 | 6 | 1.00 | -- | -- | 1.00 | -- | -- |
| Medium | 5 – 7 | 46 | 10 | 1.63 | 0.55 | 4.86 | 1.42 | 0.44 | 4.53 |
| High | 8+ | 25 | 7 | 2.10 | 0.64 | 6.94 | 1.45 | 0.37 | 5.68 |
| *p linear trend* |  |  |  | *p=0.217* |  |  | *p=0.581* |  |  |
| ***Arterial lactate, mmol/L (Median)*** |  |  |  |  |  |  |  |  |  |
| Low (reference) | <= 1.6 | 75 | 10 | 1.00 | -- | -- | 1.00 | -- | -- |
| High | 1.7+ | 41 | 13 | 1.91 | 0.77 | 4.77 | 1.13 | 0.41 | 3.10 |
| ***Arterial lactate mmol/L (Tertiles)*** |  |  |  |  |  |  |  |  |  |
| Low (reference) | <= 1.3 | 43 | 5 | 1.00 | -- | -- | 1.00 | -- | -- |
| Medium | 1.4 - 2.0 | 39 | 8 | 1.76 | 0.53 | 5.85 | 1.52 | 0.41 | 5.55 |
| High | 2.1+ | 34 | 10 | 2.53 | 0.79 | 8.10 | 1.38 | 0.37 | 5.08 |
| *p linear trend* |  |  |  | *p=0.116* |  |  | *p=0.670* |  |  |
| ***CPR, mg/L (Median)*** |  |  |  |  |  |  |  |  |  |
| Low (reference) | <= 206.5 | 62 | 8 | 1.00 | -- | -- | 1.00 | -- | -- |
| High | 206.6+ | 54 | 15 | 2.15 | 0.85 | 5.47 | 1.76 | 0.64 | 4.79 |
| ***CRP, mg/L (Tertiles)*** |  |  |  |  |  |  |  |  |  |
| Low (reference) | <= 150.2 | 41 | 6 | 1.00 | -- | -- | 1.00 | -- | -- |
| Medium | 150.3 – 251.9 | 40 | 6 | 1.02 | 0.30 | 3.45 | 0.95 | 0.26 | 3.49 |
| High | 252.0+ | 35 | 11 | 2.15 | 0.72 | 6.40 | 2.06 | 0.63 | 6.77 |
| *p linear trend* |  |  |  | *p=0.154* |  |  | *p=0.209* |  |  |

ORa: Odds ratio adjusted by age, sex, SOFA score and diagnosis of severe sepsis or septic shock.
