# Supplementary material for: Cortisol and adrenal androgens as independent predictors of mortality in septic patients
Source: PLoS One. 2019 Apr 4;14(4):e0214312. doi: 10.1371/journal.pone.0214312 (PMC6448869; doi:10.1371/journal.pone.0214312)
Supplement: S8 Table — (DOC) [file pone.0214312.s008.doc]

**S8 Table. Crude and adjusted o**dds ratios for the rest of biomarkers and severity scores on the risk of all-cause 90-day mortality.

|  |  | **Survivors** | **Non survivors** | |  |  |  |  |  |
| --- | --- | --- | --- | --- | --- | --- | --- | --- | --- |
| **Variables** | ***Cut-off points*** | ***N=108*** | ***N=31*** | ***OR*** | ***(95%*** | ***CI)*** | ***ORa*** | ***(95%*** | ***CI)*** |
| ***APACHEII (Median)*** |  |  |  |  |  |  |  |  |  |
| Low (reference) | <= 18 | 62 | 14 | 1.00 | -- | -- | 1.00 | -- | -- |
| High | 19+ | 46 | 17 | 1.64 | 0.73 | 3.66 | 0.94 | 0.38 | 2.30 |
| ***APACHEII (Tertiles)*** |  |  |  |  |  |  |  |  |  |
| Low (reference) | <= 14 | 44 | 5 | 1.00 | -- | -- | 1.00 | -- | -- |
| Medium | 15 – 20 | 32 | 13 | 3.57 | 1.16 | 11.04 | 1.90 | 0.55 | 6.61 |
| High | 21+ | 32 | 13 | 3.57 | 1.16 | 11.04 | 1.53 | 0.42 | 5.63 |
| *p linear trend* |  |  |  | *p=0.03* |  |  | *p=0.649* |  |  |
| ***SOFA* (Median)*** |  |  |  |  |  |  |  |  |  |
| Low (reference) | <= 6 | 69 | 16 | 1.00 | -- | -- | 1.00 | -- | -- |
| High | 7+ | 39 | 15 | 1.66 | 0.74 | 3.72 | 1.11 | 0.46 | 2.71 |
| ***SOFA *(Tertiles)*** |  |  |  |  |  |  |  |  |  |
| Low (referene) | <= 4 | 42 | 9 | 1.00 | -- | -- | 1.00 | -- | -- |
| Medium | 5 – 7 | 43 | 13 | 1.41 | 0.55 | 3.65 | 1.14 | 0.41 | 3.14 |
| High | 8+ | 23 | 9 | 1.83 | 0.64 | 5.24 | 1.05 | 0.31 | 3.49 |
| *p linear trend* |  |  |  | *p=0.258* |  |  | *p=0.927* |  |  |
| ***Arterial lactate, mmol/L (Median)*** |  |  |  |  |  |  |  |  |  |
| Low (reference) | <= 1.6 | 60 | 13 | 1.00 | -- | -- | 1.00 | -- | -- |
| High | 1.7+ | 48 | 18 | 1.73 | 0.77 | 3.88 | 1.06 | 0.44 | 2.57 |
| ***Arterial lactate mmol/L (Tertiles)*** |  |  |  |  |  |  |  |  |  |
| Low (reference) | <= 1.3 | 39 | 9 | 1.00 | -- | -- | 1.00 | -- | -- |
| Medium | 1.4 - 2.0 | 39 | 8 | 0.89 | 0.31 | 2.54 | 0.67 | 0.22 | 2.09 |
| High | 2.1+ | 30 | 14 | 2.02 | 0.77 | 5.30 | 1.07 | 0.36 | 3.18 |
| *p linear trend* |  |  |  | *p=0.143* |  |  | *p=0.849* |  |  |
| ***CRP, mg/L (Median)*** |  |  |  |  |  |  |  |  |  |
| Low (reference) | <= 206.5 | 60 | 10 | 1.00 | -- | -- | 1.00 | -- | -- |
| High | 206.6+ | 48 | 21 | 2.62 | 1.13 | 6.10 | 2.23 | 0.91 | 5.47 |
| ***CRP, mg/L (Tertiles)*** |  |  |  |  |  |  |  |  |  |
| Low (reference) | <= 150.2 | 41 | 6 | 1.00 | -- | -- | 1.00 | -- | -- |
| Medium | 150.3 – 251.9 | 35 | 11 | 2.15 | 0.72 | 6.40 | 2.17 | 0.68 | 6.93 |
| High | 252.0+ | 32 | 14 | 2.99 | 1.03 | 8.65 | 3.02 | 0.97 | 9.40 |
| *p linear trend* |  |  |  | *p=0.044* |  |  | *p=0.058* |  |  |

ORa: Odds ratio adjusted by age, sex, SOFA score and diagnosis of severe sepsis or septic shock.
